# Effect of inter-aural temporal envelope differences on inter-aural time difference thresholds for amplitude modulated noise

**DOI:** 10.1590/2317-1782/20232022261

**Published:** 2024-02-02

**Authors:** Vibha Kanagokar, Hasna Fathima, Jayashree Sunil Bhat, Arivudai Nambi Pitchai Muthu

**Affiliations:** 1 Department of Audiology and Speech Language Pathology, Kasturba Medical College, Mangalore, Manipal Academy of Higher Education, Manipal, Karnataka, India.; 2 Department of Audiology and Speech-Language Pathology, National Institute of Speech and Hearing - Trivandrum, Kerala, India.; 3 Kasturba Medical College Hospital - Ambedkar Circle, Mangalore, India.; 4 Department of Audiology, All India Institute of Speech and Hearing - Mysore, Karnataka, India.

**Keywords:** Inter-Aural Time Difference, Modulation Depth, Inter-Aural Modulation Depth Difference, Modulation Frequency, Envelope

## Abstract

**Purpose:**

The inter-aural time difference (ITD) and inter-aural level difference (ILD) are important acoustic cues for horizontal localization and spatial release from masking. These cues are encoded based on inter-aural comparisons of tonotopically matched binaural inputs. Therefore, binaural coherence or the interaural spectro-temporal similarity is a pre-requisite for encoding ITD and ILD. The modulation depth of envelope is an important envelope characteristic that helps in encoding the envelope-ITD. However, inter-aural difference in modulation depth can result in reduced binaural coherence and poor representation of binaural cues as in the case with reverberation, noise and compression in cochlear implants and hearing aids. This study investigated the effect of inter-aural modulation depth difference on the ITD thresholds for an amplitude-modulated noise in normal hearing young adults.

**Methods:**

An amplitude modulated high pass filtered noise with varying modulation depth differences was presented sequentially through headphones. In one ear, the modulation depth was retained at 90% and in the other ear it varied from 90% to 50%. The ITD thresholds for modulation frequencies of 8 Hz and 16 Hz were estimated as a function of the inter-aural modulation depth difference.

**Results:**

The Friedman test findings revealed a statistically significant increase in the ITD threshold with an increase in the inter-aural modulation depth difference for 8 Hz and 16 Hz.

**Conclusion:**

The results indicate that the inter-aural differences in the modulation depth negatively impact ITD perception for an amplitude-modulated high pass filtered noise.

## INTRODUCTION

In everyday conversations, the listener must often focus on the source of interest by filtering out the surrounding interfering noise. The human auditory system can correctly identify the direction of the sound source by making use of the inter-aural time difference (ITD) and the inter-aural level difference (ILD) cues which play an important role in horizontal localization^(1)^ and spatial release from masking^(2)^. This ITD and ILD information is conveyed by the temporal envelope (ENV) and temporal fine structure (TFS). The ENV refers to the amplitude fluctuations present in the signal which is depicted in terms of the short-term rate of neural firing in the auditory system and the TFS refers to the signal's rapid frequency variations which are depicted by the synchronized phase locking of the neurons^(3)^. At low frequencies, the ITD coding relies both on TFS and ENV. However, at higher frequencies, the ITD and ILD are coded by the ENV^(4)^.

The relative importance of ITD and ILD cues for spatial perception depends on various factors. Listeners tend to rely majorly on the ITD than the ILD cue since the ILD cue is found to be more degraded in adverse listening conditions^(5)^. Normal hearing individuals predominantly rely on the TFS ITD than the ENV ITD^(6)^ for horizontal localization and spatial release from masking. However, listeners may have to rely on the ENV for decoding ITD in scenarios where the TFS is not available or is poorly represented. For example, most sound coding strategies of cochlear implants encode only the ENV and discard the TFS. Also in individuals with cochlear hearing loss^(7)^ and auditory neuropathy^(8)^ the ENV ITD plays an important role since the TFS coding is affected.

The ITD derived from the ENV is however not robust and is affected by various factors related to the stimulus such as the carrier frequency, modulation frequency, modulation depth, envelope slope, binaural coherence, environmental factors such as background noise and reverberation, and/or subject-related factors^(9-12)^. The presence of background noise and reverberation is found to affect the ENV by changing its overall shape. Reverberation affects the onset gradient, slope, and modulation depth of the ENV whereas noise reduces the modulation depth of the target ENV by filling in the dips. Physiological studies have also shown that noise severely degrades ENV coding in the auditory system^(13)^.

Previous research on envelope-based ITD using high pass filtered noise bands had reported that deeper modulation depth would yield better ITD thresholds^(14-16)^. The envelope enhancement by deepening modulation depth, steeper slopes, and improved binaural coherence has shown improvement in ITD perception in CI users^(15,16)^. However, the effect of inter-aural modulation depth difference on ITD is not well understood. Preliminary evidence for the existence of such an effect was reported in a study by Pitchaimuthu et al.^(17)^ using amplitude-modulated vowel-consonant-vowel (VCV) tokens with varied inter-aural modulation depth differences in six normal-hearing young adults. The inter-aural modulation depth was kept at 100% in both ears for the reference condition and smeared by 29% and 50% in the left ear in the other two conditions. The study findings showed that an increase in the inter-aural modulation depth difference resulted in the worsening of ITD thresholds.

The perception of ITD cues is accurate when the auditory image has similar spectro-temporal characteristics in both ears, a phenomenon which is known as binaural coherence^(9,11,18)^. The ITD is an important cue for spatially segregating the desired speech signal from other competing signals^(19)^. However, the binaural coherence of the auditory input might affect the ITD-encoding^(18)^. The binaural coherence of the envelope shape is affected under certain listening conditions, such as when the target and the masker are spatially separated. Also, the level-dependent compression and bandpass filtering implemented in hearing devices will reduce the modulation depth to a different degree in both ears. The effect of inter-aural modulation depth difference on ITD threshold for speech stimuli was demonstrated by Pitchaimuthu et al.^(17)^. However, the study was performed on a small sample of six subjects. Inter-subject variability in ITD thresholds among normal hearing individuals have been reported in the literature^(20)^. In the present study, the effect of inter-aural modulation depth difference on the ITD threshold for a high pass filtered amplitude-modulated noise having a modulation frequency of 8 Hz and 16 Hz is investigated in eighteen normal-hearing young adults. The use of amplitude modulated noise stimuli instead of speech stimuli allows to study modulation specific effects with the help of a sinusoidal modulator. In addition to the three interaural modulation depth differences used in the study by Pitchaimuthu et al.^(17)^, the current study used two more interaural modulation depth differences with smaller step sizes.

## METHODS

### Participants

Eighteen young adults (age range 18- 30 years) participated in the study. All the participants had hearing thresholds ≤20 dB HL across the audiometric octave frequencies. None of the participants had any history of middle ear pathology or other neurological deficits. The study was approved by the Institutional Ethics Committee (Ref No: IEC KMC MLR 12-18/503). Written consent was obtained from all participants before the study. The procedure was carried out in a sound-treated room.

### Signal processing

ITD threshold for an amplitude modulated high pass noise was estimated as a function of the modulation depth difference between the ears. A 500 ms broadband noise with a sampling frequency of 44100 Hz was generated using MATLAB R2020b. This broadband noise was high pass filtered at 2000 Hz using a 6^th^ order Butterworth filter and modulated using an 8 Hz and 16 Hz sinusoid. The modulation depth was smeared in the left ear by a factor of 10%, 20%, 30%, 40%, and 50% resulting in 90%, 80%, 70%, 60%, and 50% of the original modulation depth. Thus, for each frequency, five inter-aural modulation depth difference conditions were created viz. C1, C2, C3, C4, and C5. C1 (modulation depth of 90% in right and 90% in left), C2 (modulation depth of 90% in right and 80% in left), C3 (modulation depth of 90% in right and 70% in left), C4 (modulation depth of 90% in right and 60% in left), and C5 (modulation depth of 90% in right and 50% in left). There were hence five waveform conditions for 8 Hz and 16 Hz and ten conditions in total. The inter-aural time difference was introduced to the waveform by applying phase-shift to left ear stimuli.

### Threshold tracking procedure

The ITD threshold was estimated using a three-interval three alternative forced-choice (3I3AFC) method for each waveform condition. The selection of waveform conditions was random. For each waveform condition, the ITD threshold was estimated twice and the average of the ITD obtained for trials 1 and 2 was considered for final analysis. The 3I3AFC method consisted of three intervals in each trial and only one interval was having the ITD cue with the lag. The participants were instructed to identify this lag interval wherein the stimulus lateralized either to the right or the left ear. The other two intervals were not having the ITD cue and hence the stimulus was perceived in the midline. The lag interval was randomly assigned. Following each trial, a response window appeared on the screen in which the participant can select the appropriate number corresponding to the lag interval. A familiarization task involving 30 trials with a 400 µsec ITD in the lag condition was carried out before the actual experiment.

The threshold tracking procedure always began with an ITD of 400 µsec and this delay was adaptively varied using the transformed 2-down 1-up procedure. The ITD decreased by a factor of 1.1 following two consecutive positive responses and increased by a factor of 1.1 following a single negative response. A total of 10 reversals were administered and the midpoint of the last 8 reversals was averaged to obtain the ITD thresholds. The stimuli were presented sequentially through headphones (Sennheiser HD280 Pro) routed via a Motu 16A audio interface.

### Data analysis

Statistical analysis was performed using SPSS. Each participant's ITD threshold was estimated as a geometric average of the last eight reversals of the transformed up-down procedure. Friedman test was used to investigate the main effect of inter-aural modulation depth differences on ITD thresholds. Wilcoxon’s signed-rank test was used for pairwise comparisons between the conditions.

## RESULTS

**Figure 1:**
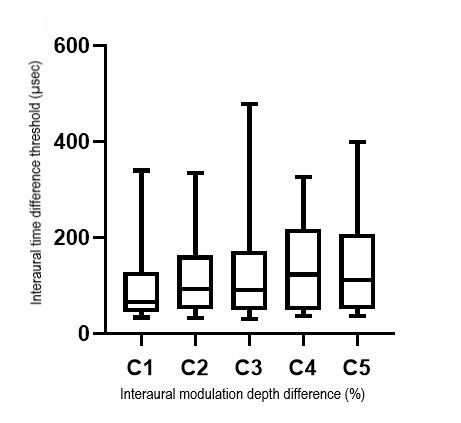
Boxplot representing the ITD thresholds for each inter-aural modulation depth difference condition for a modulation frequency of 8 Hz

**Figure 2:**
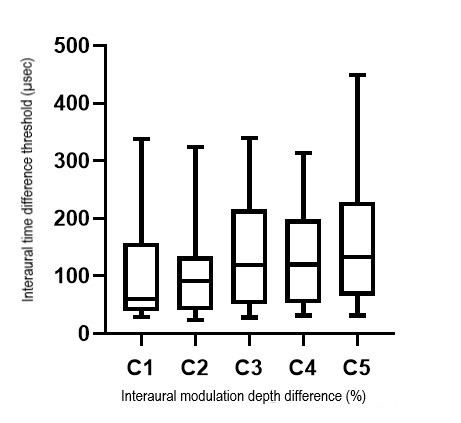
Boxplot depicting the ITD thresholds for each inter-aural modulation depth difference condition for a modulation frequency of 16 Hz

The Friedman test findings revealed a statistically significant difference in the ITD threshold with an increase in the inter-aural modulation depth difference for 8 Hz (χ^2^(4) = 10.444, *p* = 0.034) and 16 Hz (χ^2^(4) = 17.022, *p* = 0.002). The results suggest that ITD thresholds for both modulation frequencies differ significantly with an increase in the inter-aural modulation depth difference. Figure 1 and Figure 2 show the boxplot comparing the ITD thresholds for each inter-aural modulation depth difference condition for a modulation frequency of 8 Hz and 16 Hz respectively. Median (IQR) ITD threshold (µsec) for C1, C2, C3, C4, and C5 were 65.37, 93.38, 91.42, 123.23, and 112.25 respectively for 8Hz and 60.46, 91.19, 119.48, 120.37, and 132.78 for 16 Hz as shown in Figure 1 and 2.

Post hoc analysis with Wilcoxon signed-rank tests was conducted. The p values were corrected using Benjamini and Hochberg procedure for false discovery rate. For 8 Hz, there were no significant differences between C1 and C2 (Z=-1.328, p=0.184), C2 and C3 (Z=-0.501, p=0.616), C2 and C4 (Z=-1.764, p=0.078), C2 and C5 (Z=-1.285, p=0.199), C3 and C4 (Z=-1.023, p=0.306), C3 and C5 (Z=-1.241, p=0.215), C4 and C5 (Z=-0.152, p=0.879) and C1 and C3 (Z=-2.025, p=0.043). However, there was a statistically significant difference between C1 and C4 (Z=-2.983, p=0.003), and C1 and C5 (Z=-2.373, p=0.018). For 16 Hz, the ITD thresholds did not differ significantly between C1 and C2 (Z=-.152, p=0.879), C1 and C4 (Z=-1.764, p=0.078), C3 and C4 (Z=-.283, p=0.777), C2 and C4 (Z=-1.938, p=0.053), and C3 and C5 (Z=-1.807, p=0.071). However, there was a statistically significant difference between C1 and C3 (Z=-2.504, p=0.012), C1 and C5 (Z=-2.722, p=0.006), C2 and C3 (Z=-2.199, p=0.028), C2 and C5 (Z=-2.722, p=0.006), and C4 and C5 (Z=-2.069, p=0.039).

### Effect of modulation frequency

The ITD thresholds obtained with modulation frequencies of 8Hz and 16 Hz were compared to investigate the effect of modulation frequency on ITD thresholds. Wilcoxon Signed-Ranks test showed that the ITD thresholds obtained for the two modulation frequencies did not show a statistically significant difference between each other for C1(Z=-0.497, p=0.619), C2 (Z=-1.823, p=0.068), C3 (Z=-.118, p=0.906), and C5(Z=-0.166, p=0.868). For C4 (Z=-2.107, p=0.035), the ITD thresholds obtained for the two modulation frequencies showed a statistically significant difference between each other.

In addition, the inter aural cross correlation function is computed for each condition as a function of lag using a MATLAB function for normalized cross-correlation. The formula for the normalized cross-correlation is described in Equation 1.


$$Normalized\;correlation\;(R_{xy,coeff}\left(m\right))=\;\frac{R_{xy}\left(m\right)}{\sqrt{R_{xx}\left(0\right)R_{yy}\left(0\right)}}$$ (1)

**Figure 3:**
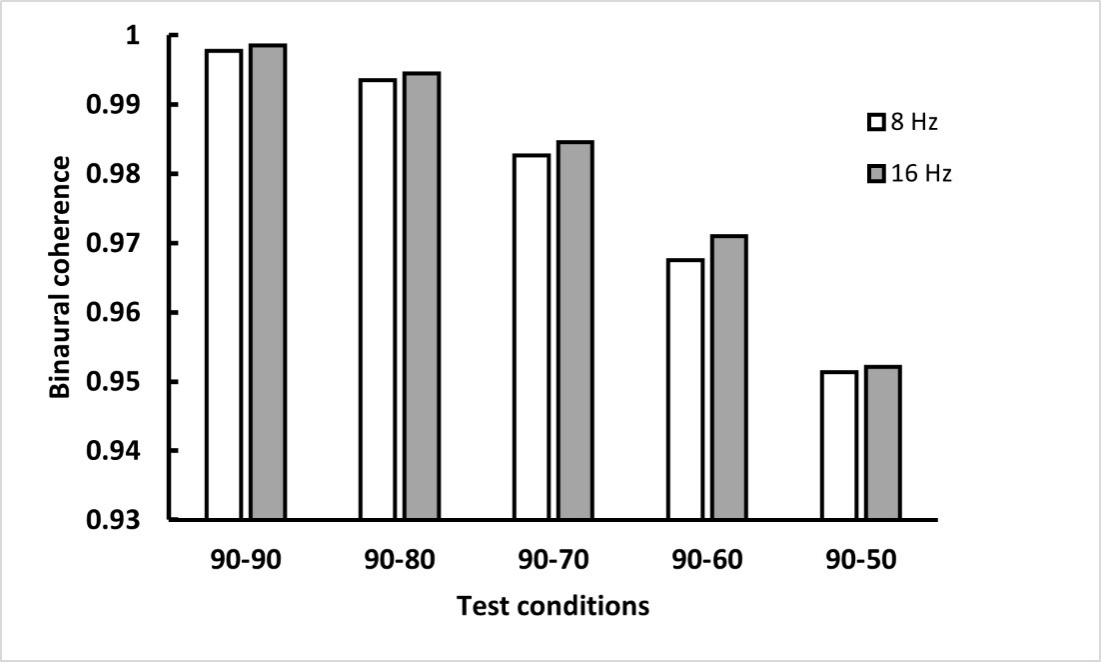
Bar graph representing the binaural coherence calculated for the interaural modulation depth difference conditions for modulations frequencies of 8 Hz and 16 Hz

Where x= right ear envelope, y= left ear envelope, N=greater of the length of x or y, m=1,2….2N-1, R_xy_ (m) represents cross correlation, R_xx_ and R_yy_ represents autocorrelation at zero lag. Binaural coherence is estimated as the maximum value of the cross-correlation function. Figure 3 shows the binaural coherence computed for interaural modulation depth difference conditions of C1, C2, C3, C4, and C5 for 8 and 16 Hz modulation frequencies. It can be seen that interaural modulation depth difference from 0% to 40% leads to reduction in binaural coherence for the noise stimuli modulated by 8 Hz and 16 Hz.

## DISCUSSION

The study investigated the effect of inter-aural modulation depth differences on the ITD thresholds for an amplitude-modulated high pass filtered noise modulated at frequencies of 8 Hz and 16 Hz. The inter-aural differences in the modulation depth were found to increase the ITD thresholds, however, increasing the modulation frequency from 8 Hz to 16 Hz did not result in any significant change in the ITD thresholds except for the condition, C4. We used two different modulation frequencies in the current study so that we have a better understanding of the effect these modulation frequencies can have on ITD thresholds if a speech stimulus is used. As the peak frequency of the speech modulation spectra is predominantly at the low frequencies^(21-23)^ we have restricted the modulation frequencies to 8 and 16 Hz.

The findings of the present study are in consonance with a previous study by Pitchaimuthu et al.^(17)^ wherein vocoded VCV consonants were used in a similar experimental paradigm in which a worsening of ITD thresholds was observed with the increase in the modulation depth difference between the two ears. Preservation of binaural coherence has been considered an important factor in ITD perception in earlier studies. For example, Monaghan et al.^(10)^ altered the binaural coherence and found that the reduced binaural coherence affected ITD perception. Goupell et al.^(24)^ who studied ITD perception using vocoded pulse trains emphasized that inter-aurally correlated envelopes were essential for maximizing the benefits of bilateral hearing devices. Earlier research^(18,25)^ has pointed out the importance of the inter-aural similarity of the envelope for performing inter-aural comparisons by ITD-sensitive neurons in the brainstem nuclei.

Several factors in the environment are shown to affect the binaural coherence by causing irregularities in the encoding of the temporal envelope of the acoustic signal. Surrounding noise and reverberation changes the overall envelope shape^(12)^. The spatial location of the masker also influences the interaural envelope differences in modulation depth. For instance, when the location of the interfering stimuli is away from the midline, its effect is more pronounced on the envelope of the stimuli in the nearer ear compared to the farther ear which results in inter-aural modulation depth difference. In addition to the environmental factors, subjective factors such as hearing loss also play a role in preserving the coherence of the signal envelope. Individuals with cochlear pathology also rely mainly on the cues provided by the ENV fluctuations for ITD estimation since the auditory system fails to code the fast frequency fluctuations. However, the presence of hearing loss reduces their ability to make use of these ENV fluctuations accurately^(26)^. Though there have been advances in hearing aid and cochlear implant technology these individuals still possess problems in binaural auditory tasks such as horizontal localization and speech understanding in noise^(27)^. This can be attributed to the fact that the compression and the band-pass filtering mechanism employed in the signal processing algorithms reduce the envelope modulation depth and onset gradients thereby affecting the coherence^(5)^.

The findings of the study has implications in improving the binaural benefits in bilateral hearing devices such as cochlear implants. In the cochlear implants, the interaural cues are encoded by the envelope cues. Studies have reported that ILD cues are insufficient for SRM and there is improvement in SRM when ITD cues are faithfully represented^(28)^. Studies on envelope enhancement have reported that modulation depth is an important envelope parameter that determine encoding of binaural cues^(15,16)^. The present study findings point to the need for improving the binaural coherence of envelope characteristics for better representation of ITD.

## CONCLUSION

The findings of the current study show that the inter-aural differences in envelope modulation depth negatively impact ITD perception of the amplitude-modulated high pass filtered noise. The preservation of the inter-aural similarity of the temporal envelope is essential for precise encoding of the ITD cues.
